# Epidemiological Profile of Patients With Tibial Fractures Treated With the Ilizarov External Fixator

**DOI:** 10.7759/cureus.87508

**Published:** 2025-07-08

**Authors:** Vinicius Rodrigues Pasetto, Igor Henrique Morais, Fernando Ferraz Faria

**Affiliations:** 1 Orthopedics and Traumatology, Cajuru University Hospital, Curitiba, BRA

**Keywords:** circular fixator, consolidation, external fixator, ilizarov, ilizarov ring fixator, tibial fracture

## Abstract

Background

Tibial fractures are common, severe, and usually result from medium or high-energy trauma. Due to their subcutaneous location, they are often open fractures. The treatment impacts quality of life and can be done using a plaster cast, external fixator, plate, or intramedullary nail. This study aims to analyze cases treated with the Ilizarov circular fixator.

Methodology

This was an epidemiological, descriptive, cohort study conducted at Cajuru University Hospital from February 2023 to December 2024 among patients with tibial fractures treated with the Ilizarov ring fixator. Acute fracture cases with complete data records were included, excluding deformities, non-unions, dynamization, or the use of a foot plate. Epidemiological variables, AO classification, type of fracture, fixator assembly, time to weight-bearing, consolidation, and removal of the device were analyzed. Consolidation was defined by radiographic signs of three cortical bones, assessed by two orthopedists.

Results

Of the 42 patients treated, 21 met the criteria. Men were predominant (90.5%), with an average age of 44 years. Closed fractures were more frequent (76.2%), with AO 41C type being the most common (33.3%). Traffic accidents were the main mechanism (61.9%). The standard fixator assembly consisted of three rings and three proximal and distal elements. Partial weight-bearing began, on average, at four weeks. Consolidation occurred in 5.5 months, and the fixator remained in place for an average of 289 days.

Conclusions

The Ilizarov ring fixator is an effective, versatile, and minimally invasive device that provides stability for both diaphyseal and articular fractures, resulting in satisfactory healing and early load-bearing, further demonstrating clinical applicability.

## Introduction

Tibial fractures are relatively common and are recognized as potentially serious and disabling injuries [1], as they usually result from moderate or high-energy trauma. Their incidence can vary in the literature depending on the time period, country, and cultural context. An average incidence of 16-18 per 100,000 inhabitants has been estimated. In the population aged 10-40 years, high-energy mechanisms are common in males, while the incidence of high-energy trauma in females is significantly lower [2].

When evaluating the fracture, AO classification is the most widely used [2-4], maintaining the predominance of simple line types. Concerning the energy of the trauma, another important assessment is the soft tissue injury, as the tibia is predominantly subcutaneous and has a high frequency of open fractures compared to other bones [4,5].

The treatment of these fractures directly affects the patient’s quality of life after the injury. The prognosis of this injury is directly influenced by the initial displacement, fragmentation, and soft tissue injury [5]. Among the possible devices are immobilization with a plaster cast, modular external fixators, ring external fixators (Ilizarov) [6], osteosynthesis with plates and screws, and intramedullary nails [5], each with its particularities and indications that must be individualized for each case.

This study aims to provide a descriptive epidemiological analysis of cases treated with the Ilizarov ring external fixator for tibial fractures, including all its portions.

## Materials and methods

This descriptive, epidemiological, cohort study analyzed medical records and radiographs of patients who underwent surgery between February and December 2023, with clinical and radiological follow-up until December 2024.

The records included patients with tibial fractures who were admitted to the Cajuru University Hospital (Curitiba, PR) and who received Ilizarov fixation as definitive treatment. The records did not include patients with incomplete information or who had inaccurately filled data of all the indicators of interest of the study, as well as those who had used Ilizarov to treat non-acute injuries, such as deformities and non-union, and those who had undergone dynamization [7] to treat the injuries, or had used a foot plate in the external fixator.

Based on the data collected by the researchers, the following characteristics were analyzed: gender; age; classification of the injuries (according to the AO classification); closed or open fractures; number of rings used in the fixator; number and type of fixation elements (olive wires or Schanz pins) and their location (proximal or distal to the fracture); average time of load-bearing initiation between metaphyseal and diaphyseal fractures; average time of fracture healing; and average time until removal of the fixator.

During patient follow-up, serial anteroposterior (AP) and lateral radiographs were obtained, primarily at two, three, four, five, and six months post-surgery, and presented randomly to two orthopedic trauma specialists to determine whether there had been bone healing. Both considered fractures that showed radiographic signs of bone union in at least three cortical bones to be consolidated.

This study was approved by the Ethics and Research Committees of Cajuru University Hospital and Pontifical Catholic University of Parana (approval number: CAAE 61312522.3.0000.0020). The data obtained were organized and processed in Microsoft Office Excel 2021, analyzed using simple frequency, mean, median, mode, and percentage values, and then presented in the text and tables.

## Results

A total of 42 patients were treated with the Ilizarov external fixator during the study period (2023), of whom 18 were excluded after the dynamization process during treatment, and three were excluded due to non-union, resulting in a final sample of 21 patients who met the inclusion and exclusion criteria. In the final sample, only patients treated with the Ilizarov external fixator for acute tibial fractures in the traditional way, without dynamization of the fixation, underwent surgery between February and December 2023.

Of these patients, 19 (90.47%) were men and 2 (9.52%) were women. The average age of the patients at the time of the trauma was 44 years (range = 22-76 years), with the average male age being 43 years (range = 22-76 years), and the average female age being 55 years (range = 46-65 years), as shown in Table 1.

**Table 1:** Description of male and female patients and open and closed fractures.

Epidemiological factors	(n/%)/(minimum-maximum)
Male	19/90.5%
Female	2/9.5%
Average age, males	43.42 (22-76)
Average age, females	55.5 (46-65)
Exposed fractures	5/23.8%
Closed fractures	16/76.2%

Among the fractures, 5 (23.8%) were open fractures and 16 (76.2%) were closed fractures. When evaluating the fractures according to AO classification, type 41C was the most frequent with 7 (33.33%) cases, followed by 42A with 4 (19.05%) cases. Table 2 describes the frequency of fractures according to the AO classification and their percentage.

**Table 2:** AO classifications, frequencies, and percentages of the study population.

AO classification	Frequency	Percentage
41A	3	14.29%
41B	0	0.00%
41C	7	33.33%
42A	4	19.05%
42B	1	4.76%
42C	1	4.76%
43A	3	14.29%
43B	0	0.00%
43C	2	9.52%

The trauma mechanisms were diverse, with 13 cases involving traffic accidents (motorcycle collision or fall, bicycle fall, or being run over), totaling 61.9% of the fractures. Three cases were considered low-energy trauma, of which two were sprain mechanisms (one refracture) and one fall from the same level after a seizure. Other mechanisms included falls from an elevated position, heavy objects falling on the limb, assault with a wooden bar, and firearm injuries.

**Figure 1:**
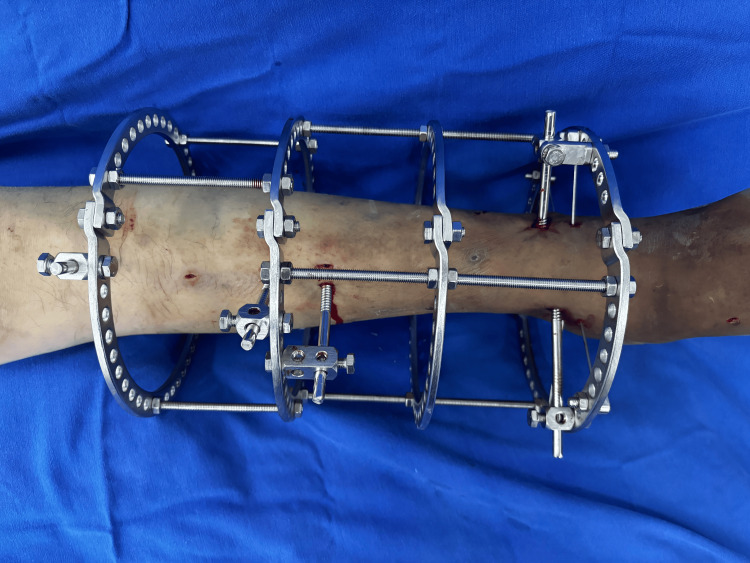
Ilizarov ring fixator with four rings. Three Shanz pins in the proximal fragment. Two Shanz pins and two olive wires in the distal fragment.

Regarding the mounting of the external fixator, the mode of rings was 3 (range = 3-5). Mounting varied according to the number of fixation elements used for each fixator configuration and each type of fracture. Among the fixation elements, wires and Schanz pins were used. The mode between the fixation elements proximal and distal to the fracture site was also 3 (range = 3-6). Table 3 lists the evaluation of the fixator configuration between the fixation elements. Figure 1 shows an example of the assembly of an external fixator.

**Table 3:** Number of fixation elements used between rings, olive wires, and Schanz pins proximal and distal to the fracture.

Description	Mode (minimum-maximum)
Number of rings	3 (3-5)
Proximal fasteners	3 (3-6)
Proximal Olive wires	2 (0-4)
Proximal Schanz pins	2 (0-4)
Distal fixation elements	3 (3-6)
Distal Olive wires	0 (0-4)
Distal Schanz pins	3 (1-5)

When correlating diaphyseal (AO 42) and metaphyseal (AO 41 and 43) fractures with the mode of proximal and distal fixation elements, the mode was three fixation elements proximal and three distal to the fracture. Notably, the range of fixation elements used in metaphyseal fractures varied more, reaching six proximal fixation elements and six distal elements depending on the fracture, as shown in Table 4.

**Table 4:** Correlation between the location of the fractures and the type of fixation elements used for fixation.

Type of fracture	Proximal fixation mode (minimum-maximum)	Distal fixation mode (minimum-maximum)
Diaphyseal fractures (AO 42)	3 (3-4)	3 (3-3)
Metaphyseal fractures (AO 41 and 43)	3 (3-6)	3 (3-6)

The average time for patients to start partial weight-bearing was four weeks. Time to load start varied according to the location of the fracture and is shown in Table 5, with the average time for each location as well as its highest and lowest values.

**Table 5:** Average time to partial load-bearing (in weeks) and fracture location.

AO fracture type	Time in weeks until weight-bearing (minimum-maximum), SD
41	5.20 (0-12), ±4.34
42	0.00 (0-0), ±0.00
43	7.20 (0-14), ±5.76

Among the patients who achieved healing by the December 2024 evaluation, the average time to healing was 5.5 months (range = 2-8 months). In the analysis of the length of time the fixator was used, the average time until the synthesis material was removed was 289 days (range = 112-539 days).

## Discussion

This study aimed to analyze the profile of patients undergoing treatment with the Ilizarov external fixator to better understand the patient characteristics and treatment methods.

In the literature, men account for approximately two-thirds of all cases, aged around 32 years [2]. We found that around 90% of fractures occurred in men with a higher average age. Females accounted for 36% of fractures with an average age of 48 years [2], differing from the women treated with the Ilizarov technique, who were older (on average, 55 years) and in a lower proportion (9%). The incidence of fractures in males peaks between the ages of 10-20 years and can reach 43.5 per 100,000 inhabitants [2].

AO type A fractures have been described as the most frequent of all fracture types, followed by types B and C [2,3], representing the fact that the energy of the trauma progressively increases and the trauma becomes more severe, but less common. Among the subtypes, there is a divergence in the data between 42-A1 and 42-A3 [2,3]. In contrast, the fracture most frequently treated with Ilizarov was 41C, followed by 42A, 41A, and 43A, respectively. This difference is related not only to the severity of the fracture associated with extensive multifragmentation but also to the location, soft tissue injury, risk of infection, and future complications.

According to the literature, the frequency of open fractures is around 25% [3], which can vary according to the trauma mechanism, ranging from punctiform wounds to lacerations that put the limb’s prognosis at risk. Among open fractures, almost two-thirds can be classified as Gustilo III, followed by Gustilo I and II in that order [3]. There was a correlation between the increased severity of AO lesions and wounds classified by Gustilo.

We found a similar proportion of open fractures (23.8%) and believe that the vast majority of cases were associated with a high Gustilo classification, including 3B and 3C. This is based on our trauma service routine, which tends to treat simple fractures with exposures up to Gustilo 3A with internal fixation and preferably with an intramedullary tutor, whenever possible, on patient admission.

Another important factor assessed was the time taken to initiate partial weight-bearing, which includes “touch support” or “proprioceptive touch.” This assessment varied according to the fracture site (metaphyseal and diaphyseal) and the fracture line. We observed a routine of early weight-bearing for patients with diaphyseal fractures, as all 42A fracture patients started some kind of weight-bearing in the immediate postoperative period. In metaphyseal injuries, the indication for starting load-bearing varied according to the joint involvement of the case, allowing load-bearing earlier for extra-articular and partial joint fractures, and avoiding it for a longer time in complete joint injuries.

Although the Ilizarov ring external fixator is known for its use in correcting deformities and bone defects, its use and superiority have been established in fractures where open reduction with internal fixation and treatment with plaster casts do not perform well, especially in juxta-articular, multifragmented, complex, or highly exposed fractures [8].

Classic fixator assembly involves the use of rings and wires, but Schanz pins have also been used to secure the structure to the bone. The use of complete rings instead of half rings is known to increase the stability of the system. Another important concept is the tensioning of the olive wires to provide the fixator sufficient rigidity. Of note, tensioning is important, especially when the wires have an angle of around 90° between them. Moreover, fixation involving only wires is less rigid than the hybrid assembly with the addition of Schanz pins [8,9].

Descriptions of fixator assembly show the use of two rings per bone segment [8]. Our patients were often treated with three rings (range = 3-5 rings). This may have influenced the extended duration of fixator use and the longer time until healing.

No fixator was assembled using only olive wires or Schanz pins. Both elements were used in all fractures at some stage. The use of Schanz pins in the distal fragment was predominant, probably to add stability to the more mobile segment of the fracture.

The median healing time found in our study was 5.5 months, with a range of two to eight months in cases that achieved healing. This is comparable to data in the literature, which shows a median healing time of 23 weeks (range = 18-32 weeks) [10].

Some of the results differed from the literature on tibial fractures and their particularities. Many of these differences were because this service is a referral center for orthopedics and traumatology, where severe cases are more frequently treated. Given this, we still have to consider that the majority of tibial fractures with less complex trauma received either an intramedullary nail or open reduction with internal fixation in both the proximal and distal metaphysis and were not included in this evaluation, even though they are the major proportion of the tibial fractures treated at our hospital.

The main limitation of this study is the small number of patients selected, justified by the selection of cases within a timeframe of 10 months. We understand that a larger number of patients should be selected and that the parameters assessed could be more comprehensive, with the objective of better understanding and treating patients with tibial fractures.

## Conclusions

The Ilizarov external fixator is an effective and versatile method for different types of tibial fractures. It is minimally invasive and capable of providing sufficient stability for both diaphyseal and articular fractures. Bone consolidation was achieved within an acceptable timeframe, with early weight-bearing in most cases. Hence, this method is applicable and reproducible in various clinical settings.
